# Harmony in Life Scale - Turkish version: Studies of validity and reliability

**DOI:** 10.1186/s41155-017-0073-9

**Published:** 2017-09-04

**Authors:** Seydi Ahmet Satici, Emine Gocet Tekin

**Affiliations:** 1 Department of Psychological Counseling and Guidance, Faculty of Education, Artvin Coruh University, 08000 Artvin, Turkey; 2 Foreign Languages Department, Sakarya University, 54187 Adapazarı, Sakarya Turkey

**Keywords:** Harmony, Well-being, Quality of life, University students, Scale adaptation

## Abstract

This article presents the adaptation and psychometric evaluation of the Turkish version of Harmony in Life Scale (Turkish-HiL). The present paper investigates (study 1; *N*_*1*_ = 253) confirmatory factor analysis, measurement invariance; (study 2; *N*_*2*_ = 231) concurrent validity; (study 3; *N*_*3*_ = 260) convergent and known-group validities; (study 4; *N*_*t − t*_ = 50) test-retest, Cronbach alpha, and composite reliabilities of the Turkish-HiL. In study 1, based on a confirmatory factor analysis, results confirmed that unidimensional-factor structure. The results suggested that the model demonstrated a configural and metric invariance across the gender groups. In study 2, Turkish-HiL significantly correlated with measures of satisfaction with life, subjective happiness, positive affect, and negative affect. In study 3, Turkish-HiL was predicted positively by flourishing, conversely, negatively predicted by depression, anxiety, and stress. Finally, in study 4, alpha, composite and test-retest reliabilities are acceptable. Overall, the scale presented here may prove useful for satisfactorily assessing, in Turkish, the harmony in life of the university students.

## Background

Humans feel the desire to live in a social environment as a whole. Harmony described as “friendly or cooperative relations among people, such that social interactions are congruous and conflict free” (American Psychological Association (APA), 2015, p. 483). Harmony is the key to living in balance with their own environment. Balance and flexibility are considered to be important virtues in harmonizing different aspects within the world (Li, 2008). Therefore, the nature of harmony in itself is relational in which things flourish through mutual support and dependence (Li, 2008). According to psychological well-being perspective, the concept of harmony is accepted as a holistic entity which emphasizes balance and flexibility considering social and environmental issues.

Harmony involves different values and judgments according to the culture where they belong. Therefore, it is important to learn about cultural values for a better understanding of harmony. In that respect, the models proposed by Hofstede (1980) and Markus and Kitayama (1991) have been discussed briefly. Hofstede’s (1980) cultural value systems classification is the most referred to a cultural variable. Despite its popularity in the 1980s, recent researches have shown contradictory results. The cultural value dimension of individualism-collectivism is considered to include different goals and routes for self-development (Greenfield, 1994; Markus, Mullally, & Kitayama, 1997).

Markus and Kitayama (1991) proposed a model of the self in which self-cognitions are divided into two distinct components: independent and interdependent self. According to the independent model, specifically in Western cultures, the self is assumed to be an independent, bounded, and unique entity, in which the primary aim of the behavior is to influence other individuals or environmental factors in line with one’s own needs and goals. On the other hand, according to the interdependent model, the self is characterized as interdependent and connected entity in which the primary aim of the behavior is to regulate one’s own needs and goals in harmony with the expectations of others. According to Markus and Kitayama (1991), independent self-construal is common in Western (especially North American) cultures; whereas, interdependent self-construal is more common in non-Western cultures such as parts of Asia, Africa, and Central and South America. In the case of the independent model, the main priority is given to autonomy to achieve happiness (Campbell, 1981; Diener & Diener, 1995). On the contrary, in the case of the independent model, harmony is viewed as the main concern in achieving happiness (Nisbett, 2003). Maintaining harmonious social relations and working out what is good for the group is likely to take precedence over establishing superiority or uniqueness.

### Harmony in life

Harmony is basically related to subjective well-being with slight differences in the assessment of well-being. Subjective well-being (SWB) as one of the most extensively studied concepts in positive psychology is defined as how a person evaluates his or her own life with respect to four separate aspects: life satisfaction, positive affect, the absence of negative affect, and domain satisfaction (Diener, Suh, Lucas, and Smith; 1999). Positive affect and negative affect refer to the amount of pleasant and unpleasant feelings that people experience in their lives and assess the affective component of SWB, while satisfaction with life and domain satisfaction are considered as the cognitive components of SWB, evaluating beliefs about the quality of one’s life.

According to Diener (1994), satisfaction with life refers to the judgmental process in which individuals compare their perceived life circumstances with their self-imposed standards that are unique for each person. However, Kjell and colleagues (2013) argued that this conceptualization might characterize a distinct and limited view of cognitive well-being as the criterion of satisfaction is enforced by the researcher himself. Seeing life satisfaction as *one* important aspect of cognitive well-being rather than an overarching or superior concept, Kjell and colleagues (2013) proposed that harmony in life can complement satisfaction with life. Harmony in life refers to “a global and overall assessment of whether one’s life involve balance, mindful non-judgmental acceptance, fitting in and being attuned with one’s life” (Garcia et al., 2014).

The development of harmony in life is based upon the cognitive aspects of psychological functioning as altruistic mental commitment (Dambrun & Ricard 2011), fit focused secondary control (Morling & Evered 2006), non-judgmental, mindful adaptation (e.g., see Kabat-Zinn 2004), and mental resilience (Kashdan & Rottenberg 2010). Dambrun and Ricard (2011) postulated a theoretical model in which well-being is linked to two qualitatively distinct aspects of the psychological functioning of the self. They argued that perceiving the self as a fixed and independent entity causes a self-centered psychological functioning which causes one to approach gratifying things and experiences and to avoid those that are unpleasant or threatening. Conversely, a selfless psychological functioning emerges when the self is seen as flexible and strongly connected with others and with all of the elements in the environment. Morling and Evered (2006) conceptualized secondary control as fit focused involving two key aspects “adjustment of the self” and “acceptance of the environment”; in other words, the secondary control includes both accepting the situation as it is and adjusting the self to fit that situation. They argued that these principles underlying secondary control are conducive to well-being since they promoted feelings of relatedness (Morling et al., 2002).

Unlike life satisfaction, harmony in life involves concepts such as mindfulness and psychological flexibility. Kjell et al. (2013) argued that current life satisfaction overemphasizes the judgmental process of evaluating the surroundings according to one’s expectations which can lead to feelings of incompleteness and dissatisfaction. Mindfulness involves accepting internal and external experiences as they occur without judging or elaborating on that experience (Kabat-Zinn, 1994). Psychological flexibility, on the other hand, conceptualized as a regulation process of adapting to changing situational demands, re-organizing mental resources changing point of view, and maintaining balance between desires, needs, and life domains (Kashdan & Rottenberg, 2010). Both mindfulness and psychological flexibility related to harmony in life, in that, they both emphasize that human beings have the potential to adjust harmoniously to all of the elements of the environment.

### Turkish culture

Since this study was conducted on a Turkish sample, it can be useful to mention some features of Turkish culture. As it was mentioned above, the cultural value dimension of individualism-collectivism (I–C) is considered to include different goals and routes for self-development (Greenfield, 1994; Markus, Mullally, & Kitayama, 1997). Studies regarding cultural values of Turks revealed that Turkish people, particularly among the better-educated segments of the society, tend to have more individuated self-construal while maintaining their tendencies for relatedness (Imamoglu & Karakitapoglu-Aygun, 2004). Aygun and Imamoglu (2002) reported that there was a strong tendency towards both individuation and relationality, with a decrease in relatedness among the better-educated classes of the Turkish society. Likewise, in a study among Turkish and Euro-Canadian samples of university students, Uskul, Hynie, and Lalonde (2004) found that the two cultural groups did not differ on independent self-construal; indeed, Turkish women were found to have more independent self-construal than both Turkish men and Canadian men and women. More recently, Satıcı (2016) stated that the top-rated sources of happiness that were mentioned most frequently among Turkish university students were reflecting both collectivistic values such as being loved/knowing that you are loved, spending time with others, and individualistic values such as making lots of money. In the same study, low-rated resources of happiness were also reported to manifest both collectivistic (mediation) and individualistic (aloneliness, being rewarded) values. Therefore, it can be suggested that Turkish culture seems to integrate both collectivistic and individualistic orientations in itself.

In this study, we aimed to adapt the Harmony in Life Scale (HiL; Kjell et al., 2016) into Turkish and test the psychometric properties. The study will ensure a valid and reliable measure for the evaluation of harmony in life in the Turkish language to be used to understand harmony in Turkish culture. In the present investigation, four separate studies were conducted for the adaptation of the Turkish-HiL. Study 1 investigated the factor structure of the Turkish-HiL. Study 2 investigated the concurrent validity of the Turkish-HiL. Convergent validity and known-group validity of the Turkish-HiL was investigated in study 3. Finally, the reliability of the Turkish-HiL was examined in study 4.

## Study 1. Validity: factor structure

The original Harmony in Life Scale from Kjell and colleagues (2016) was translated into Turkish by five experts using the parallel blind technique, and then it was back-translated to assure the accuracy of the translation with the source version (Behling & Law, 2000). The translated versions of the scale were discussed with seven doctoral students. The final version was agreed upon by the authors of the current study. After the translation of the scale into the Turkish language, the construct validity of the scale was examined by confirmatory factor analysis.

### Method

#### Participants

Turkish university students [*N* = 253, 135 females, 118 males, *M*_age_ = 20.23 years, SD = 1.93) completed the Turkish-HiL on a voluntary and anonymous basis. Of the participants, 64 (25%) were first-year students, 99 (39%) were second-year students, 42 (17%) were third-year students, and 48 (19%) were fourth-year students. All participants were recruited in their classroom and were asked to fill out the paper-based questionnaires. Completion of the scale required no more than 3 min.

#### Procedure

Responses to the five-item Turkish-HiL questionnaire were subjected to a confirmatory factor analysis (CFA) using maximum likelihood estimation procedure. CFA was also performed on female and male data independently to test the plausibility of differing factor structures related to gender. Invariance analyses were conducted in order to examine gender difference in the Turkish version of Harmony in Life Scale. Configural, metric, scalar, and strict invariance analyses were evaluated. Comparative fit index (CFI), root mean square error of approximation (RMSEA), standardized root mean square residual (SRMR), and goodness of fit index (GFI) were used as fit statistics. The ratio of chi-square to degrees of freedom was also examined.

### Result

The items of the Turkish-HiL were checked for skew (*S* ranged −1.28 to −0.79) and kurtosis (*K* ranged −0.20 to 1.43), which were within the normal range. After the seen normal distribution, we conducted the CFA. The theoretical factor structure of the HIL was confirmed by a confirmatory factor analysis. Standardized loadings, standard errors, *t* values, and *R*^*2*^ values are shown in Table 1.

**Table 1:** CFA results of Turkish-HiL

Item	λ	θ	*t*	*R* ^*2*^
Yaşam tarzım benim çevremle uyum içerisinde olmama olanak sağlar.	.61	.62	9.92	.38
Yaşamımın birçok yönü denge içindedir.	.77	.40	13.09	.60
Bir uyum içerisindeyim.	.84	.29	14.61	.71
Yaşamımdaki farklı koşulları kabul ederim.	.46	.79	7.05	.21
Çevreme iyi bir şekilde uyum sağlarım.	.50	.75	7.74	.25

*Note.* All scholar can use Turkish-HiL for scientific purposes without written permission; λ = standardized factor loadings; θ = error variance

As can be seen in Table 1 factor loadings for Turkish-HiL ranged from .46 to .84. The Turkish-HiL provided fit well across the total sample, an acceptable fit to the data, *χ*^2^_(5, *N* = 253)_ = 9.01, *p* < .05; CFI = .99; GFI = .99; IFI = .99; SRMR = .033; RMSEA = .056. The following gender difference analyses were based on 135 female participants’ and 118 male participants’ Turkish-HiL score. Table 2 shows the Goodness-of-Fit Indices for confirmatory models of total, separate, and invariance.

**Table 2:** Fit indexes of Turkish-HiL

	χ ^2^	df	*∆χ* ^*2*^	*∆df*	*p*	CFI	IFI	GFI	SRMR	RMSEA
Total sample	9.01	5	–	–	–	.99	.99	.99	.033	.056
Separate analysis										
Male	12.34	5	–	–	–	.94	.94	.96	.062	.081
Female	4.09	5	–	–	–	1.00	1.00	.99	.028	.001
Invariance										
Configural invariance	58.04	15	–	–	–	.91	.91	.91	.122	.151
Metric invariance	61.15	19	3.11	4	> .05	.91	.91	.90	.069	.133
Scalar invariance	132.50	28	71.35	9	< .001	.74	.74	.85	.187	.172
Strict invariance	133.49	29	.99	1	> .05	.75	.74	.85	.163	.169

When the Turkish-HiL model was fitted separately to the samples of male and female, the results showed relatively similar and acceptable fit. Configural and metric invariance models except for RMSEA indicated a good fit. Scalar and strict invariance models show slightly lower and non-acceptable fit to the data. Guidelines suggested by Cheung and Rensvold (2002) were relied on in this study. The results suggested that the model demonstrated a configural, metric invariance across the gender groups. However, the lack of scalar invariance indicated that Turkish-HiL is not adequate for gender comparisons, especially considering the huge drop of the CFI.

## Study 2. Validity: concurrent validity

In this study, the concurrent validity of the Turkish-HiL was investigated. The association between satisfaction with life, subjective happiness, and positive-negative affective subjective well-being and Turkish-HiL was examined to establish the concurrent validity. “Positive affect + life satisfaction – negative affect” formula was used to assess subjective well-being.

### Method

#### Participants

Two hundred thirty-one (123 females, 108 males; *M*_age_ = 19.73 years, SD = 1.82) undergraduate students from two different universities in the Middle and Northwestern region of Turkey. Of the participants, 71 (31%) were first-year students, 55 (24%) were second-year students, 59 (26%) were third-year students, and 46 (20%) were fourth-year students. The scales were administered in classrooms after informed consents were obtained from the participants’. Completion of the scale required no more than 15 min.

#### Measures

In addition to Turkish-HiL, the Positive and Negative Affect Scale, the Satisfaction with Life Scale, and Subjective Happiness Scale were also included in this study. The detailed information about the instruments is given below.

##### Positive and Negative Affect Scale (PANAS; Watson, Clark, & Tellegen, 1988)

The PANAS consists of 10 affective adjective words, and positive affect words such as “inspired,” “interested,” and “strong” and negative affect words such as “afraid,” “nervous,” and “irritable” have been used respectively. Participants answered to which degree they felt each of the affects on a scale from (1) “very slightly or not at all” to (5) “extremely.” The PANAS adapted to Turkish by Gençöz (2000) from the original English version. Cronbach’s alpha reliabilities for positive and negative affect were respectively .83 and .86 (Gençöz, 2000). In the present study, Cronbach’s alpha coefficients were .83 and .79 for the positive and negative affect, respectively.

##### The Satisfaction with Life Scale (SWLS; Diener, Emmons, Larsen, & Griffin, 1985)

The SWLS consists of five items (e.g., My life conditions are excellent) using 7-point Likert-type response format, ranging from 1 (strongly disagree) to 7 (strongly agree). Possible scores range from 5 to 35 with higher scores reflecting a greater level of life satisfaction. The SWLS adapted to Turkish by Durak, Senol-Durak, and Gencoz (2010) from the original English version. The Turkish-SWLS has good reliability with Cronbach’s alpha .81 and validity with confirmatory factor analysis (χ^2^/df = 2.026, IFI = .994, TLI = .987, CFI = .994, SRMR = .020, and RMSEA = .043; Durak et al., 2010). In the present study, Cronbach’s alpha coefficient was .80.

##### The Subjective Happiness Scale (SHS; Lyubomirsky & Lepper 1999)

The SHS consists of four items (e.g., In general, I consider myself) using 7-point Likert-type response format, ranging from 1 (very unhappy) to 7 (very happy). Possible scores range from 4 to 28 with higher scores reflecting a greater level of life satisfaction. The SHS adapted to Turkish by Akin and Satici (2011) from the original English version. The Turkish-SHS has good reliability with Cronbach’s alpha .86 and validity with confirmatory factor analysis (RMSEA = .000, NFI = .99, CFI = 1.00, IFI = 1.00, RFI = .98, GFI = 1.00, AGFI = .99, and SRMR = .015; Akin & Satici, 2011). In the present study, Cronbach’s alpha coefficient was .70.

#### Procedure

Correlation analysis was conducted to the associations between concurrent variables scores and Turkish-HiL scores as a means to show that harmony in life, measured via the Turkish-HiL, exhibited theoretically or conceptually expected relationships with certain variables (e.g., subjective well-being, life satisfaction, subjective happiness, positive affect, and negative affect), as outlined in the literature. Also, the 95% confidence interval (CI) for the Turkish-HiL was calculated. If the upper and lower limits of the confidence interval do not include 0, we can say that there is a statistically significant difference between the means of the groups.

### Results

Concurrent validity of the Turkish-HiL was examined in this part of the study. Confidence interval and correlations of the Turkish-HiL with other well-being measures assessed are shown in Table 3.

**Table 3:** Correlations of The Turkish-HiL and the Other Well-Being Scales with Confidence Intervals

Well-being measures	Turkish-HiL	
	*r*	95% CI
Satisfaction with life	.44^***^	.33–.54
Subjective happiness	.43^***^	.32–.53
Subjective well-being	.51^***^	.41–.60
Positive affect	.35^***^	.23–.46
Negative affect	−.31^***^	−.42 to −.19

*N* = 231; ^***^p < .001

As predicted, the Turkish-HiL was associated positively with life satisfaction (*r* = .44), subjective happiness (*r* = .43), positive affect (*r* = .35), and subjective well-being (*r* = .51). On the other hand, the Turkish-HiL was negatively associated with negative affect (*r* = −.31), (all *p*s < .001).

## Study 3. Validity: convergent and known-group validity

The aim of this study was to examine the convergent validity and known-group validity of the Turkish-HiL to provide additional evidence for the validity. The convergent role of depression, anxiety, stress, and flourishing on harmony in life was investigated in this study. Known-group validity is established by examining whether there are clear distinctions between depression, anxiety, and stress scales and Turkish-HiL.

### Method

#### Participant

In this study, participants (*N* = 260; 136 females, 124 males; *M*_age_ = 20.36, SD = 2.19) were recruited from the same university with study 2 but different departments. Of the participants, 71 (31%) were first-year students, 55 (24%) were second-year students, 59 (26%) were third-year students, and 46 (20%) were fourth-year students. Completion of the scale required no more than 20 min.

#### Measures

In addition to Turkish-HiL, the Depression, Anxiety and Stress Scale and the Flourishing Scale were also included in this study. The detailed information about the instruments is given below.

##### Depression Anxiety Stress Scale (DASS; Lovibond & Lovibond, 1995)

The DASS consists of 42 items with three sub-scales: depression (e.g., I found it difficult to work up the initiative to do things), anxiety (e.g., I felt scared without any good reason), and stress (e.g., I was in a state of nervous tension). Participants answered to which degree they felt each of the items on a scale from (0) “did not apply to me at all” to (3) “applied to me very much, or most of the time”. The DASS adapted to Turkish by Akin and Çetın (2007) from the original English version. Cronbach’s alpha reliabilities for depression, anxiety, and stress were .90, .92, and .92, respectively (Akin & Cetin, 2007). In the present study, Cronbach’s alpha coefficients were .81, .87 and .90 for the depression, anxiety, and stress, respectively.

##### The Flourishing Scale (FS; Diener et al., 2010)

The FS consists of eight items (e.g., I am competent and capable in the activities that are important to me) using 7-point Likert-type response format, ranging from 1 (strongly disagree) to 7 (strongly agree). Possible scores range from 8 to 48 with higher scores reflecting greater level of life satisfaction. The FS adapted to Turkish by Telef (2013) from the original English version. The Turkish-FS has good reliability with Cronbach’s alpha .80 and validity with confirmatory factor analysis (χ^2^/df = 4.65, GFI = .96, NFI = .94, RFI = .92, CFI = .95, IFI = .95, SRMR = .04, and RMSEA = .08; Telef, 2013). In the present study, Cronbach’s alpha coefficient was .89.

#### Procedure

In order to establish the convergent validity of the Turkish-HiL, regression analyses were performed with flourishing, depression, anxiety, and stress as independent variables and Turkish-HiL total score as the dependent variable. Known-group validity also examined in this step. For this purpose, means and standard deviations for depression, anxiety, and stress were calculated. The means of these variables were divided into three levels by distributing half standard deviation above the mean, half standard deviation below the mean, and the rest between upper and lower levels as moderate. One-way analysis of variance was conducted in order to determine whether depression, anxiety, and stress levels of the participants differentiate according to Turkish-HiL.

### Results

Table 4 illustrates the results of regression analysis for the convergent role of flourishing, depression, anxiety, and stress on Turkish-HiL.

**Table 4:** Regression results of convergent validity

Variables	Turkish-HiL	
	*β*	*t*
Flourishing	.55^**^	10.61^**^
Depression	−.50^**^	−9.18^**^
Anxiety	−.40^**^	−7.07^**^
Stress	−.37^**^	−6.32^**^

Note. ^**^p < .01

Regression results indicated that Turkish-HiL was predicted positively by flourishing (*β* = .55). Conversely, Turkish-HiL was negatively predicted by depression (*β* = −.50), anxiety (*β* = −.40), and stress (*β* = −.37).

**Fig. 1:**
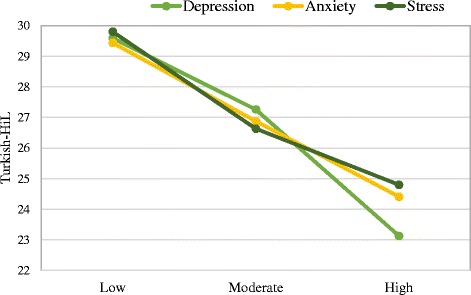
Mean levels of depression, anxiety, and stress on the Turkish-HiL for participants with low, moderate, and high levels (significant differences observed)

Results were then analyzed using a one-way analysis of variance, between-subjects design. This analysis revealed Turkish-HiL significantly differ in terms of depression, *F*_(2, 257)_ = 47.76, *p* < .001, *η*^2^ = .27, anxiety, *F*_(2, 257)_ = 23.91, *p* < .001, *η*^2^ = .16, and stress level *F*_(2, 257)_ = 24.59, *p* < .001, *η*^2^ = .16. The sample means are displayed in Fig. 1. Tukey’s HSD test showed that participants with a high level of depression, anxiety, and stress scored significantly lower on Turkish-HiL than did participants with a moderate level of depression, anxiety, and stress (all *p*s < .01) and low depression, anxiety, and stress (all *ps* < .01). Tukey’s HSD test also showed that participants with a moderate level of depression, anxiety, and stress scored significantly lower on Turkish-HiL than did participants with a low level of depression, anxiety, and stress (all *p*s < .01).

## Study 4. Reliability

Measurement instruments should be both valid and reliable for the credibility of the study. For this reason, establishing validity would demand to establish reliability**.** The aim of this study is to examine the internal consistency, composite, and test-retest reliability of the Turkish-HiL.

### Method

#### Participant

Test-retest reliability was conducted with 50 (24 females, 26 males, *M*_age_ = 19.42, SD = 1.44) participants.

#### Procedure

The Cronbach’s alpha and composite reliability coefficient was estimated for the entire study and for each study separately to assess the reliability. Test-retest reliability of the Turkish-HiL with a 6-week interval was examined.

### Result

Table 5 shows test-retest, the Cronbach’s alpha, and composite reliability coefficients for the whole/entire study and for each study respectively.

**Table 5:** Reliabilities of the Turkish-HiL

Item Number	Study I (*N*_1_ = 253)		Study II (*N*_2_ = 231)		Study III (*N*_3_ = 260)		Total data (*N*_total_ = 744)		Test-retest (*N*_test-retest_ = 50)	
	Item-total correlations (corrected)	Reliability α–CR	Item-total correlations (corrected)	Reliability α–CR	Item-total correlations (corrected)	Reliability α–CR	Item-total correlations (corrected)	Reliability α–CR	Item correlations	Total correlations
Item 1	.57	.79–.78	.58	.79–.80	.55	.77–.78	.57	.78–.78	.76	.89
Item 2	.63		.60		.57		.60		.81	
Item 3	.69		.66		.67		.67		.80	
Item 4	.44		.39		.39		.41		.77	
Item 5	.49		.63		.57		.56		.79	

Note: *α* Cronbach alpha reliability, *CR* composite reliability

Cronbach’s alpha coefficients were calculated total data as .78 for the Turkish-HiL. Cronbach’s alpha coefficients ranged between .77 and .79 for the Turkish-HiL when studies were considered separately. Composite reliabilities ranged from .78 and .80 when studies were considered separately and were calculated for total data as .78. Six-week interval test-retest reliability was found .89. As can be seen in Table 5, corrected item-total correlations ranged between .39 and .69. These findings indicated that the Turkish-HiL has an acceptable reliability.

## Discussion

The current study aimed to translate, culturally adapt, and validate the translated version of Harmony in Life Scale in a sample of Turkish university students. The data was collected from university students of two different universities in Turkey. After establishing linguistic equivalence, we investigated the structure, concurrent, convergent, known-group validities, and the reliabilities of Turkish-HiL. The Turkish-HiL showed good item homogeneity, similar to the original HiL, and good internal consistency. Moreover, the results of the confirmatory factorial analysis demonstrated acceptable fit to the data, supporting the one-factor structure of the original scale.

The association between satisfaction with life, subjective happiness, and positive-negative affect subjective well-being and Turkish-HiL was examined in order to establish the concurrent validity. As predicted, the Turkish-HiL was found to be associated positively with life satisfaction, subjective happiness, positive affect, and subjective well-being, while negatively associated with negative affect. Our findings were consistent with the findings of the original scale (Kjell et al., 2016).

The convergent validity of the flourishing, depression, anxiety, and stress over Turkish-HiL was examined. The results indicated that students with higher scores in flourishing displayed higher levels of harmony in life. Conversely, depression, anxiety, and stress negatively predicted harmony in life. The findings in our study suggested that harmony in life had positive correlations with dimensions of well-being and negative correlations with concepts related to mental health, which are in line with previous studies (Garcia et al., 2014; Kjell et al., 2016). Finally, Cronbach’s alpha, composite reliabilities, and test-retest results suggested acceptable levels of internal consistency and high levels of test-retest reliability. With regard to the known-group validity evaluation, our findings demonstrated that participants with a high level of depression, anxiety, and stress scored significantly lower on Turkish-HiL than did participants with moderate and low levels of depression, anxiety, and stress. It also revealed that participants with a moderate level of depression, anxiety, and stress scored significantly lower on Turkish-HiL than did participants with a low level of depression, anxiety, and stress.

### Limitations

Despite its significant contribution to the study of harmony in life in Turkish culture, the present study has some limitations. First, the validity and reliability of the Turkish-HiL were evaluated in a group of university students in this study; however, studies on different age groups from various backgrounds in the Turkish population are required. Additional studies are necessary to examine the applicability of Turkish-HiL in other social contexts. Second, this study relied on self-report measures for university students. Although the study had a sufficient sample size, our study lacks random assignment. The sample in this study consisted of university students who voluntarily participated in the survey, which may result in positive bias in participants (Groth-Marnat, 2003). Third, although separate analysis confirmed the Turkish-HiL model for both the males and the females, according to scalar invariance results, Turkish-HiL is not adequate for group comparisons (male × female).This non-equivalence should be taken into consideration, and future research is needed to test the scalar invariance. Finally, the cross-sectional nature of the data poses another limitation which limited our ability to establish the direction of the relationship between variables. Longitudinal studies are needed to determine these relationships.

### Recommendations for future research

The limitations of the study may provide opportunities for further studies. Future research may involve a more random sample of subjects of varying age, gender, religion, race, and education levels in order to strengthen the outcome of the study. Additionally, researchers may further investigate the psychometric properties of the Turkish-HiL by using other instruments. Furthermore, the scale’s capacity to detect and understand changes in the levels of harmony in life at both individual and population-levels has not yet been assessed. Future research may investigate the scale’s suitability for use in evaluation studies using a longitudinal design.

Finally, further research also can examine the Turkish-HiL by controlling for the influence of individualism/collectivism statistically to see if cultural differences play a significant role in developing a valid instrument.

### Implications and conclusion

The findings of our study have important implications for well-being studies in Turkey. The Turkish-HiL as an important addition to the positive psychology studies in Turkey and can be used by both practitioners and researchers. This scale can be used to investigate the well-being in respect to harmony in life and to examine the variables which promote harmony in life and, in turn, well-being.

Using Turkish-HiL together with other well-being instruments (e.g., SWLS and/or the Circles of Life and the Ladders of Life) may help researchers create a better understanding of the underlying dynamics of well-being. The instrument can be used as a tool researching and understanding the effect of Turkish-HiL on life experiences. Understanding of how harmony in life predicts general well-being will help practitioners and researchers in the development of effective interventions to increase well-being. Overall, this study has revealed that the Turkish version of the Harmony in Life Scale has acceptable psychometric properties.
